# TECHNOLOGY-ASSISTED CARE PLANNING IN PRIMARY CARE OF A PROVIDER SHORTAGE AREA

**DOI:** 10.1093/geroni/igad104.1436

**Published:** 2023-12-21

**Authors:** Mingon Kang, Laurie Kim, Ji Yoo

**Affiliations:** Howard Hughes College of Engineering at UNLV, Las Vegas, Nevada, United States; Kirk Kerkorian School of Medicine at UNLV, Las Vegas, Nevada, United States; Kirk Kerkorian School of Medicine at UNLV, Las Vegas, Nevada, United States

## Abstract

We applied faireness algorithm to identify and mitigate bias in healthcare system. We also developed DeepPlan soft that can assist primary care providers, patients and caregivers with providing real-time risk prediction system, for example, discussing nursing home placement and designation of power of attorney. Our DeepPlan was built using cloud database system than can be exchangeable in secure way between patients and primary care providers. We linked DeepPlan use to workforce education and now we are ready for presenting education and patient clinical outcomes of using DeepPlan in primary care in a provider shortage area.

